# Undenatured Type II Collagen Ameliorates Inflammatory Responses and Articular Cartilage Damage in the Rat Model of Osteoarthritis

**DOI:** 10.3389/fvets.2021.617789

**Published:** 2021-03-04

**Authors:** Cemal Orhan, Vijaya Juturu, Emre Sahin, Mehmet Tuzcu, Ibrahim Hanifi Ozercan, Ali Said Durmus, Nurhan Sahin, Kazim Sahin

**Affiliations:** ^1^Department of Animal Nutrition, Faculty of Veterinary Medicine, Firat University, Elazig, Turkey; ^2^Research and Development, Lonza, Morristown, NJ, United States; ^3^Division of Biology, Faculty of Science, Firat University, Elazig, Turkey; ^4^Department of Pathology, Faculty of Medicine, Firat University, Elazig, Turkey; ^5^Department of Surgery, Faculty of Veterinary Medicine, Firat University, Elazig, Turkey

**Keywords:** osteoarthritis, inflammation, MIA, Nf-κB, UC-II

## Abstract

Osteoarthritis (OA) is an age-related joint disease that includes gradual disruption of the articular cartilage and the resulting pain. The present study was designed to test the effects of undenatured type II collagen (UC-II®) on joint inflammation in the monoiodoacetate (MIA) OA model. We also investigated possible mechanisms underlying these effects. Female Wistar rats were divided into three groups: (i) Control; (ii) MIA-induced rats treated with vehicle; (iii) MIA-induced rats treated with UC-II (4 mg/kg BW). OA was induced in rats by intra-articular injection of MIA (1 mg) after seven days of UC-II treatment. UC-II reduced MIA-induced Kellgren-Lawrence scoring (53.3%, *P* < 0.05). The serum levels of inflammatory cytokines [IL-1β (7.8%), IL-6 (18.0%), TNF-α (25.9%), COMP (16.4%), CRP (32.4%)] were reduced in UC-II supplemented group (*P* < 0.0001). In the articular cartilage, UC-II inhibited the production of PGE2 (19.6%) and the expression of IL-1β, IL-6, TNF-a, COX-2, MCP-1, NF-κB, MMP-3, RANKL (*P* < 0.001). The COL-1 and OPG levels were increased, and MDA decreased in UC-II supplemented rats (*P* < 0.001). UC-II could be useful to alleviate joint inflammation and pain in OA joints by reducing the expression of inflammatory mediators.

## Introduction

Osteoarthritis (OA) is the most common disorder of the musculoskeletal system and the biggest cause of disability in developed and developing countries. Besides aging and joint injury, factors such as obesity, inheritance, and stress may trigger the OA formation by low-grade systemic inflammation (1). These factors cause chronic inflammation in the joints, followed by cartilage and joint space decrease (2). Increasing pro-inflammatory cytokines including interleukin 1 β (IL-1β), IL-8, and tumor necrosis factor-alpha (TNF-α) activate the nuclear factor kappa B (NF-κB) pathway that promotes the catabolic process in the joint cartilage via upregulation of matrix metalloproteinases (MMPs) (3). In OA, initial proteolytic cleavage in type II collagen, the main component of the joint cartilage, provoked by MMPs preferentially occurs between Gly^794^ and Leu^795^. After the initial cleavage, the helix form of type II collagen becomes more susceptible to enzymatic denaturation (4). Denatured fibril components of cartilage are recognized as an antigenic determinant by the immune system. These cartilage antigens cause autoimmune reactions, then increase inflammatory cytokines and other OA inducers by increasing joint inflammation and cartilage disruption (5). Therefore, osteoarthritis becomes a chronic and progressive disease that is difficult to treat.

Currently, acetaminophen, non-steroidal anti-inflammatory drugs (6), hyaluronic acid, corticosteroid (7), glucosamine, and chondroitin sulfate (8) are the most commonly used agents not only in humans but also in animals to reduce the symptoms of OA. However, these agents are inadequate to effectively reduce the pain or reverse the OA symptoms (9). Undenatured type II collagen (UC-II), a collagen product that modulates the immune system by oral tolerance, has become a novel alternative agent to support joint health over the past two decades (10). Small amounts of UC-II taken from the oral route interact with gut-associated lymphoid tissue (GALT); here, the naive T cells (T_h_0) transform into T regulatory cells (Treg) targeting the type II collagen (11, 12). When these specific Treg cells encounter type II collagen, recognized as an antigen by the immune system, they prevent autoimmune reactions by reducing killer T cell attacks on joint cartilage (10) and stimulates anti-inflammatory cytokine production (11).

Monosodium iodoacetate injection into the knee joint of rats highly imitates the symptomatic, histomorphologic, and molecular changes seen in human OA by causing chondrocyte death and glycolysis impairment (13). To the best of our knowledge, there is no mechanistic preclinical study conducted on the attenuating effect of UC-II on the MIA-induced OA in rats. Therefore, in this study, we evaluated the effects of UC-II on the inflammatory responses and oxidative stress of articular cartilage in a rat osteoarthritis model. We also tested the impact of UC-II on molecular mechanisms in OA, including NF-κB, MMP3, and receptor activator of NF-κB ligand-osteoprotegerin (RANKL-OPG).

## Materials and Methods

### Animals

Twenty-one female Wistar Albino rats (8 weeks old and weighing 170 ± 20 g) were obtained from the Firat University Experimental Research Center. The choice of the sex of the animals, that is, females, was based on the findings that autoimmune arthritis is mediated by sex hormones and that female rats are more susceptible to arthritis than males (14). Rats were housed in cages with water and feed *ad libitum*, kept at a constant temperature of 22 ± 2 °C, and controlled lighting (12 h−12 h light–dark cycle). The Firat University Animal Ethical Committee approved (182-2019/126) all procedures.

### Experimental Design

A total of 21 rats were randomly divided into three groups: (i) Control rats (*n* = 7), (ii) MIA, arthritic control rats (MIA) (*n* = 7), and (iii) MIA+UC-II, UC-II® (4 mg/BW/day) supplemented arthritic rats (*n* = 7). The OA rat model was performed, as previously described (15). To induce OA rat model, the right knee of the rats were shaved and disinfected with 70% alcohol following anaesthetization using xylazine (10 mg/kg) and ketamine hydrochloride (50 mg/kg). 1 mg of MIA (Sigma, St. Louis, USA) was dissolved in 50 μL saline and injected into right knee joints through the infrapatellar ligament using a 0.3 ml insulin syringe fitted with a 29-G needle (15). Saline (50 μl) was injected into the right knee joints through the infra-patellar ligament in the control group. Starting a week before injection with MIA, the UC-II® supplement (Lonza Consumer Health Inc, USA) was orally given daily until day 30. For rats, a dose of 4 mg BW UC-II® was calculated based on a 40 mg/kg human equivalent dose according to a previous study (16). All rats were observed every other alternate day to assess knee joint swelling. After the end of the 30-day UC-II supplementation, the rats were sacrificed, and blood and the specimens of the articular cartilage were collected for the follow-up experiment.

### Laboratory Analyses

The blood samples were centrifuged at 3.000 rpm for 10 min, and the harvested sera were kept at −80°C until the day of analysis. Serum levels of glucose, creatinine, blood urea nitrogen (BUN), total protein (TP), globulin (GLOB), alanine aminotransferase (ALT), aspartate aminotransferase (AST), alkaline phosphatase (ALP), and total bilirubin (TBIL) were measured by the automatic analyzer (Samsung Electronics, Suwon, Korea) with using rat specific kits.

Serum levels of TNF-α, IL-1β, IL-6, cartilage oligomeric matrix protein (COMP), c-reactive protein (CRP), prostaglandin E2 (PGE2), and osteocalcin (OCN) were determined with ELISA kits according to the manufacturer's instructions. The optical density of the samples was read using a microplate reader (Elx-800, Bio-Tek Instruments Inc, Vermont, USA) at a wavelength of 405 nm. A standard curve was generated using the optical density values of the standard solution for these parameters. Serum MDA concentration was measured using the fully automatic HPLC (Shimadzu, Kyoto, Japan) equipped with a pump (LC-20AD), an ultraviolet-visible detector (SPD-20A), an inertsil ODS-3 C18 column (250 × 4.6 mm, 5 m), a column oven (CTO-10ASVP), an autosampler (SIL-20A), a degasser unit (DGU-20A5) and a computer system with LC solution Software (Shimadzu, Kyoto, Japan). The activities of superoxide dismutase (SOD), catalase (CAT), and glutathione peroxidase (GSH-Px) were determined using the commercially available kits (Cayman Chemical, Ann Arbor, MI, USA) according to the manufacturer's procedure.

### Gait Test and Joint Swelling

The hind paws of the rats were brushed with dark blue ink to perform gait analysis. The rats were then allowed to walk on a white paper (60 cm-long, 7 cm-wide). A dark chamber was placed at the end of the track to entice the rats. The footprints of the rats were scanned at 300 dpi and calculated with Image J software (National Institutes of Health, USA) that was used for the analysis of the paw area (cm^2^), paw width (cm), and stride length (cm) measurements (17). After scarification, the right knee was isolated, and the femur, tibia, and patella were dissected free of muscle. The diameter (mm) was measured at least three times using a digital caliper (Mitutoyo, Kawasaki, Japan) to determine joint swelling.

### Radiographic Assessment

The deteriorations of knee joint structures were assessed by X-ray radiography that was taken under anesthesia for all rats per experimental group according to the Kellgren- Lawrence scoring system (18).

### Histopathological Analysis

After euthanasia, the right knee joints of all rats were dissected and removed from the surrounding soft tissue. The removed knee joints were fixed in 10% formalin solution for 2 days, subsequently kept in 10% nitric acid for 30 days to decalcify the inorganic matter. The knee joints were cut vertically and then embedded into paraffin blocks. The sample sections of 4 μm thickness obtained from the paraffin blocks were stained with hematoxylin-eosin (H.E.). Cartilage damages of joints were examined with light microscopy and evaluated according to the Mankin system by an expert (19).

### Western Blotting

Western blot analysis was done as defined previously (20) with minor modifications. Briefly, synovial tissue lysates were prepared in ice-cold RIPA buffer mixed with protease inhibitors. Protein content was measured by a Qubit 2.0 Fluorometer according to the manufacturer's protocol (Invitrogen, Life Technologies Corporation, Carlsbad, CA, USA). Cell lysates (30 μg protein/lane) were resolved by 12% Sodium dodecyl sulfate-polyacrylamide gel electrophoresis (SDS–PAGE), then transferred into a 0.45 um nitrocellulose membrane. The transferred proteins, blocked with bovine serum albumin to prevent unspecific interactions, were incubated overnight with the diluted (1:1,000) primary antibodies purchased from Santa Cruz Biotechnology (Dallas, TX, USA) for IL-1β, IL-6, TNF-α, interferon regulatory factor 7 (IRF7), cyclooxygenase-2 (COX-2), nuclear factor-kappa β kinase subunit gamma (IKK-γ), monocyte chemoattractant protein-1 (MCP-1), NF-κB, MMP-3, collagen type 1 (COL-1), OPG, and RANKL. Next, the blotted membranes were washed and incubated with the secondary antibody. The mouse monoclonal β-actin antibody was used (A5316; Sigma–Aldrich, USA) to control the protein loading. Specific binding between primary and secondary antibodies was visualized using diaminobenzidine. The western blotting was repeated at least three times to confirm the results for all proteins. Finally, the membranes were scanned and transferred to Image J software (National Institutes of Health, USA) for densitometric analyses.

### Statistical Analyses

The sample size was computed as seven per group using G^*^Power (Version 3.1.9.4) software, which based Cohen's d from one-way ANOVA formulas, with 0.05 type I error, 0.9 effect size, and a power of 90%. Data were presented as the mean ± standard deviation (SD). One-way ANOVA and *post hoc* Tukey HSD tests were used for the data that fits the parametric assumptions, while non-parametric data were analyzed using Mann-Whitney *U*. SPSS software version 22.0 (IBM Corp., Armonk, NY, USA) was used for all statistical analyses. At the 0.05> *p* level, statistical results were accepted as significant.

## Results

### Biochemistry and Inflammatory Parameters

OA induction with intraarticular injection of MIA did not affect the various serum biochemical parameters except ALP (Table 1). ALP levels of the MIA group was lower compared to the control and MIA+UC-II groups (*P* < 0.05). We also determined whether UC-II supplementation has any suppressive effects on the production of OA-associated inflammatory cytokines, including TNF-α, IL-6, COMP, and CRP in serum after MIA injection (Figure 1). Results reveal that OA induction significantly increased levels of serum inflammatory parameters. However, UC-II supplementation significantly reduced TNF-α, IL-6, COMP, CRP, and PGE2 levels in serum compared to the untreated MIA group (*P* < 0.001). In contrast to inflammatory parameters, intraarticular MIA injection reduced OCN levels compared to the control (*P* < 0.0001). However, UC-II supplementation in the MIA + UC-II group elevated serum OCN levels compared with untreated OA rats (*P* < 0.0001).

**Table 1:** Effect of undenatured type II collagen (UC-II) supplementation on serum biochemical analyses in monosodium iodoacetate (MIA) induced osteoarthritis rats (*n* = 7).

**Items**	**Groups**			P^*^
	**Control**	**MIA**	**MIA+UC-II**	
GLU (mg/dl)	108.00 ± 7.48	112.67 ± 11.33	108.67 ± 5.16	0.592
CR (mg/dl)	0.44 ± 0.09	0.41 ± 0.07	0.40 ± 0.09	0.768
BUN (mg/dl)	22.70 ± 2.32	23.52 ± 3.95	23.87 ± 3.49	0.825
TP (g/dl)	6.98 ± 0.18	6.73 ± 0.49	7.03 ± 0.20	0.260
ALP U/L	130.17 ± 12.7	103.67 ± 10.29^*^	131.67 ± 21.75^#^	0.012
GLOB (g/dl)	3.22 ± 0.19	3.28 ± 0.36	3.40 ± 0.14	0.456
ALT (U/L)	66.00 ± 5.87	70.50 ± 6.38	69.17 ± 5.71	0.430
AST (U/L)	111.33 ± 13.29	115.00 ± 8.17	111.83 ± 6.62	0.784
TBIL (mg/dl)	0.20 ± 0.01	0.20 ± 0.03	0.21 ± 0.02	0.504

MIA, monosodium iodoacetate; GLU, glucose; CR, creatinine; BUN, blood urea nitrogen; TP, total protein; GLOB, globulin; ALT, alanine aminotransferase; AST, aspartate aminotransferase; ALP, alkaline phosphatase; TBIL, total bilirubin. Mean values of parameters are demonstrated with ± standard deviations. ANOVA and Tukey's post hoc test were used to compare the results among different treatment groups. Statistical significance between groups is shown by

* p < 0.05 compared to the control group and

# *p < 0.05 compared to the MIA group*.

**Figure 1:**
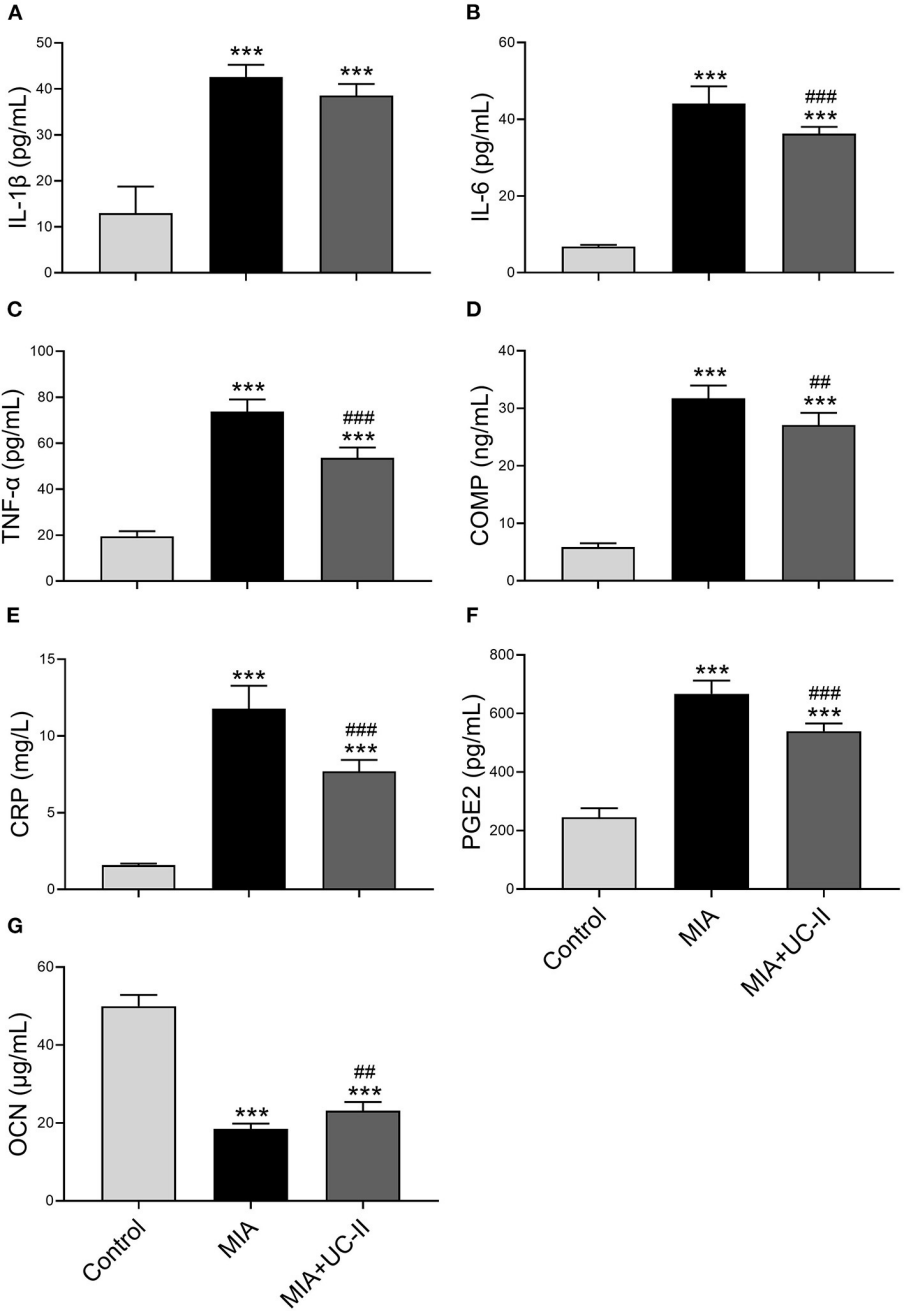
Effect of undenatured type II collagen (UC-II) supplementation on serum interleukin-1β (IL-1β; **A**), interleukin-6 (IL-6; **B**), tumor necrosis factor α (TNF-α; **C**), cartilage oligometrix matrix protein (COMP; **D**), c-reactive protein (CRP; **E**), prostaglandin E2 (PGE2; **F**), and osteocalcin (OCN; **G**) levels in monosodium iodoacetate (MIA) induced osteoarthritis rats (*n* = 7). The bars represent the mean and standard deviation of data. ANOVA and Tukey's *post-hoc test* were used to compare the results among different treatment groups. Statistical significance between groups is shown by ^***^P < 0.001 compared to the control group and ^*##*^*P* < 0.01, ^*###*^*P* < 0.001 compared to the MIA group.

### Serum Antioxidant Enzymes and MDA

**Figure 2:**
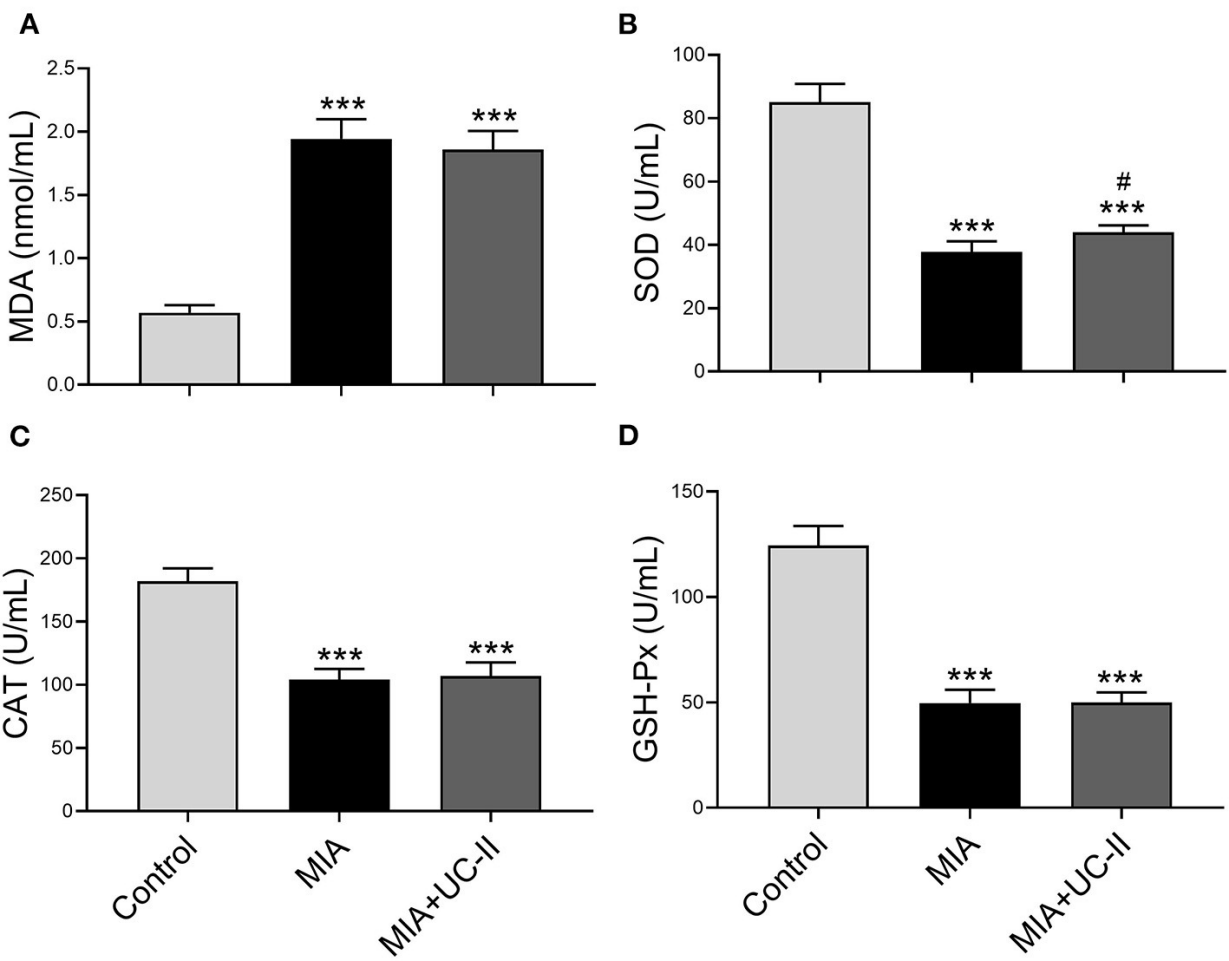
Effect undenatured type II collagen (UC-II) on serum malondialdehyde (MDA; **A**), superoxide dismutase (SOD; **B**), catalase (CAT; **C**), and glutathione peroxidase (GSHPx; **D**) levels in monosodium iodoacetate (MIA) induced osteoarthritis rats (*n* = 7). The bars represent the mean and standard deviation of data. ANOVA and Tukey's *post-hoc test* were used to compare the results among different treatment groups. Statistical significance between groups is shown by: ^***^P < 0.001 as compared to the control group and ^#^*P* < 0.05 as compared to MIA group.

OA control rats showed significantly lower serum activities of antioxidant enzymes of MIA and MIA+UC-II compared with healthy control rats (Figure 2). With the UC-II supplementation, serum SOD levels increased when compared to untreated MIA rats (*P* < 0.0001), whereas serum levels of CAT and GSH-Px, were not affected by UC-II supplementation compared to the MIA group (*P* > 0.05). Rats subjected to MIA showed a significant increase in serum MDA levels (Figure 2A). However, UC-II supplementation showed decreased MDA levels compared to untreated OA rats (*P* > 0.05).

### Gait Test and Joint Swelling Measurements

**Figure 3:**
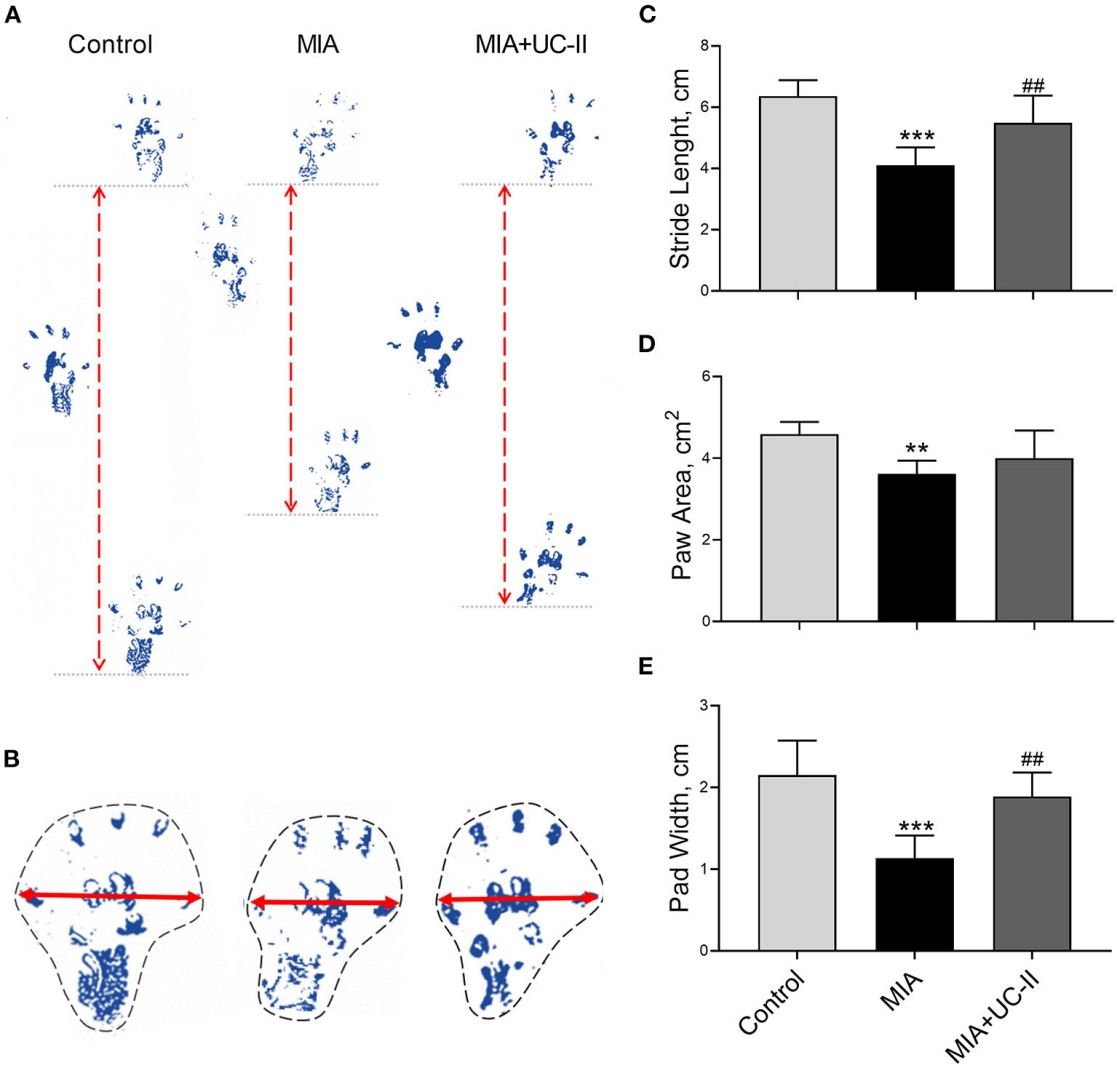
Effect undenatured type II collagen (UC-II) on stride length **(C)**, paw area **(D)**, and paw width **(E)** in monosodium iodoacetate (MIA) induced osteoarthritis rats (*n* = 7). Representative measures of stride length **(A)**, paw area **(B)** and paw width **(B)** are shown. The bars represent the mean and standard deviation of data. ANOVA and Tukey's *post-hoc test* were used to compare the results among different treatment groups. Statistical significance between groups is shown by ^**^P < 0.01, ^***^P < 0.001 compared to the control group and ## *P* < 0.01 compared to the MIA group.

**Figure 4:**
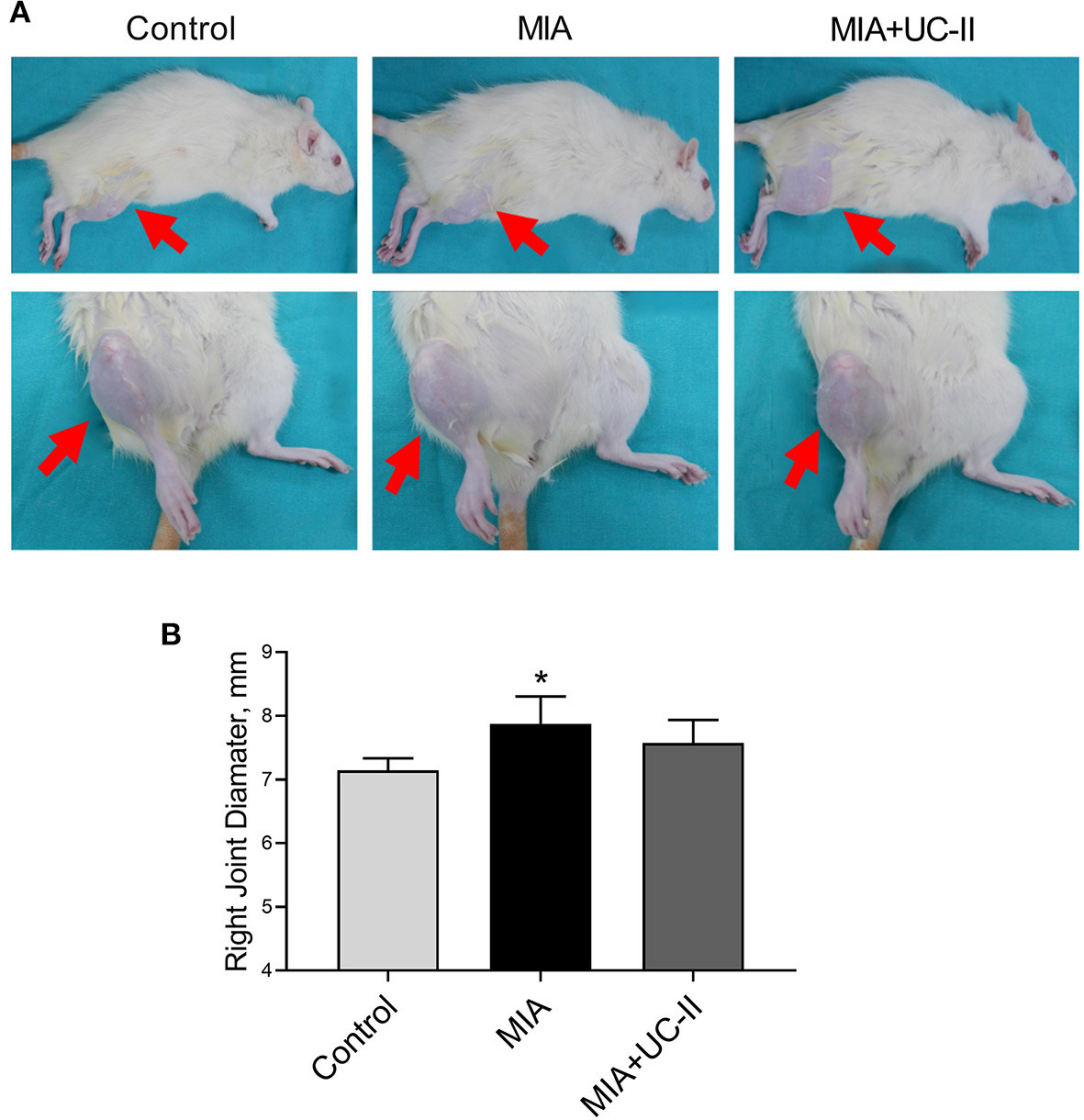
Effect of undenatured type II collagen (UC-II) on knee swelling **(A)** and right knee joint diameter **(B)** in monosodium iodoacetate (MIA) induced osteoarthritis rats (*n* = 7). The bars represent the mean and standard deviation of data. ANOVA and Tukey's *post-hoc test* were used to compare the results among different treatment groups. Statistical significance between groups is shown by: ^*^P < 0.05 as compared to the control group.

Intraarticular MIA injection in the knee joint induced the OA reduced gait stability. As shown in Figure 3, MIA treatment significantly reduced stride length (Figure 3C), paw area (Figure 3D), and paw width (Figure 3E) compared to the control groups (*P* < 0.01, for all). However, the group supplemented with UC-II increased stride length (Figure 3C), paw area (Figure 3D), and paw width (Figure 3E; *P* < 0.01). As shown in Figure 4, the right knee diameter of the MIA injected group was markedly increased compared with the control group (*P* < 0.05).

### Histopathological Analysis

**Figure 5:**
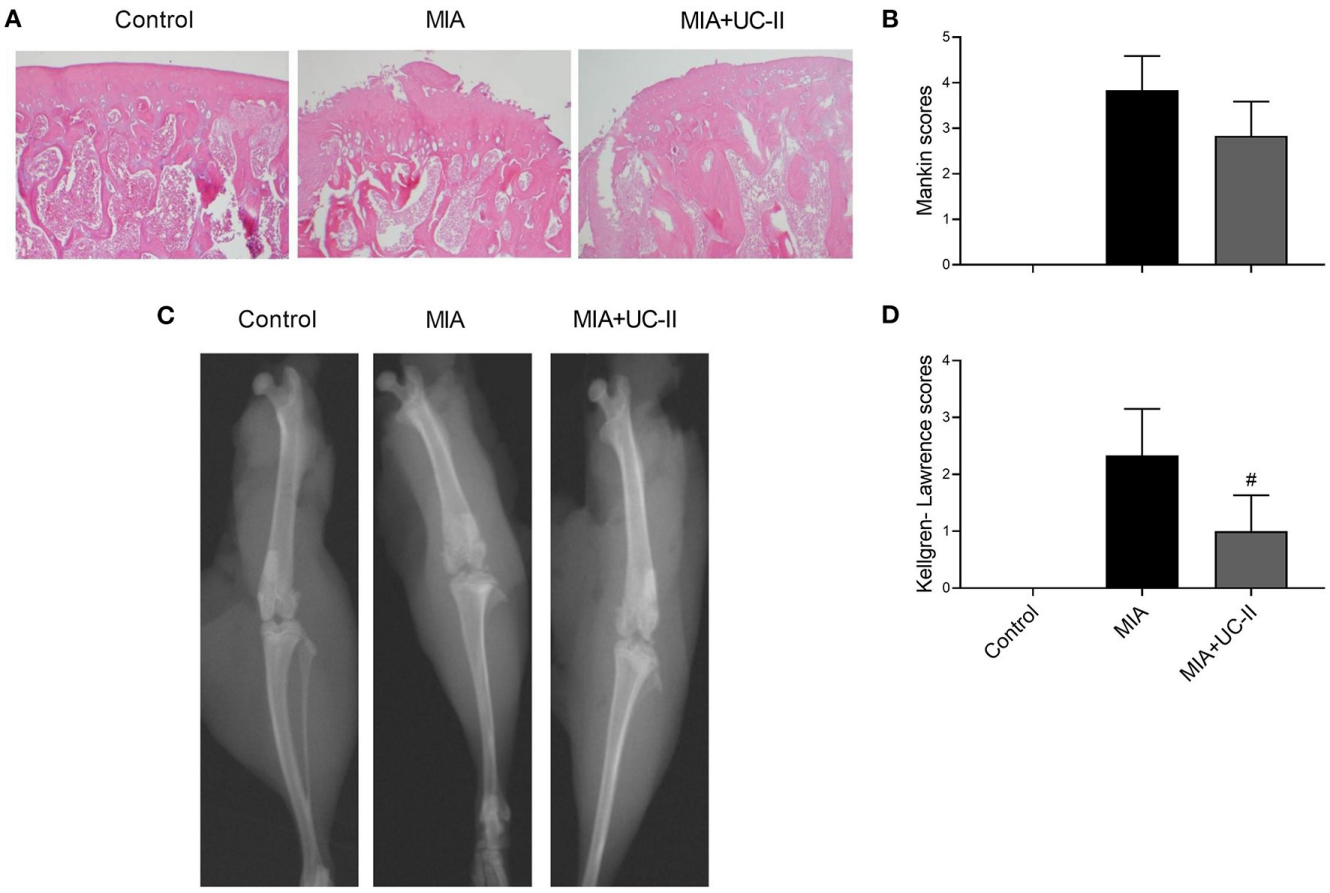
Effect of undenatured type II collagen (UC-II) on the knee joint radiographic and histopathologic findings in monosodium iodoacetate (MIA) induced osteoarthritis rats (*n* = 7). Representative radiographic **(A)** and histopathologic **(B)** images obtained at the end of the experiment are shown. Kellgren-Lawrence and Mankin scores were shown in **(C)** and **(D)**, respectively. The bars represent the mean and standard deviation of data. Mann-Whitney *U* test were used to compare the results among different treatment groups. Statistical significance between groups is shown by: ^#^*P* < 0.05 as compared to the MIA group.

The joints of the control group demonstrated a smooth articular cartilage surface with the underneath layer of flattened chondrocytes in the tangential zone, and chondrocytes were normally distributed in parallel rows transitional and radial zones of the articular cartilage and uniform cell distribution (Figure 5A). Unlike the control group, the MIA injected group showed severe surface irregularity and surface cleft, and matrix loss of the associated articular cartilage, and disappearance of chondrocytes in the cartilage (Figure 5A). UC-II supplementation attenuated the structural, morphological changes in the articular cartilages compared with those of the MIA-injected group. However, there was no statistical difference found between the MIA and MIA+UC-II groups according to the Mankin scoring system (Figure 5B; *P* > 0.05).

### Radiographic Assessment

As shown in the radiographic image in Figure 5C, intraarticular MIA injection increased Kellgren-Lawrence scoring in the knee joint of MIA rats. According to the Kellgren-Lawrence scoring system, the UC-II supplementation decreased the joint space compared to the MIA rats (*P* < 0.05; Figure 5D).

### Western Blot Analyses

**Figure 6:**
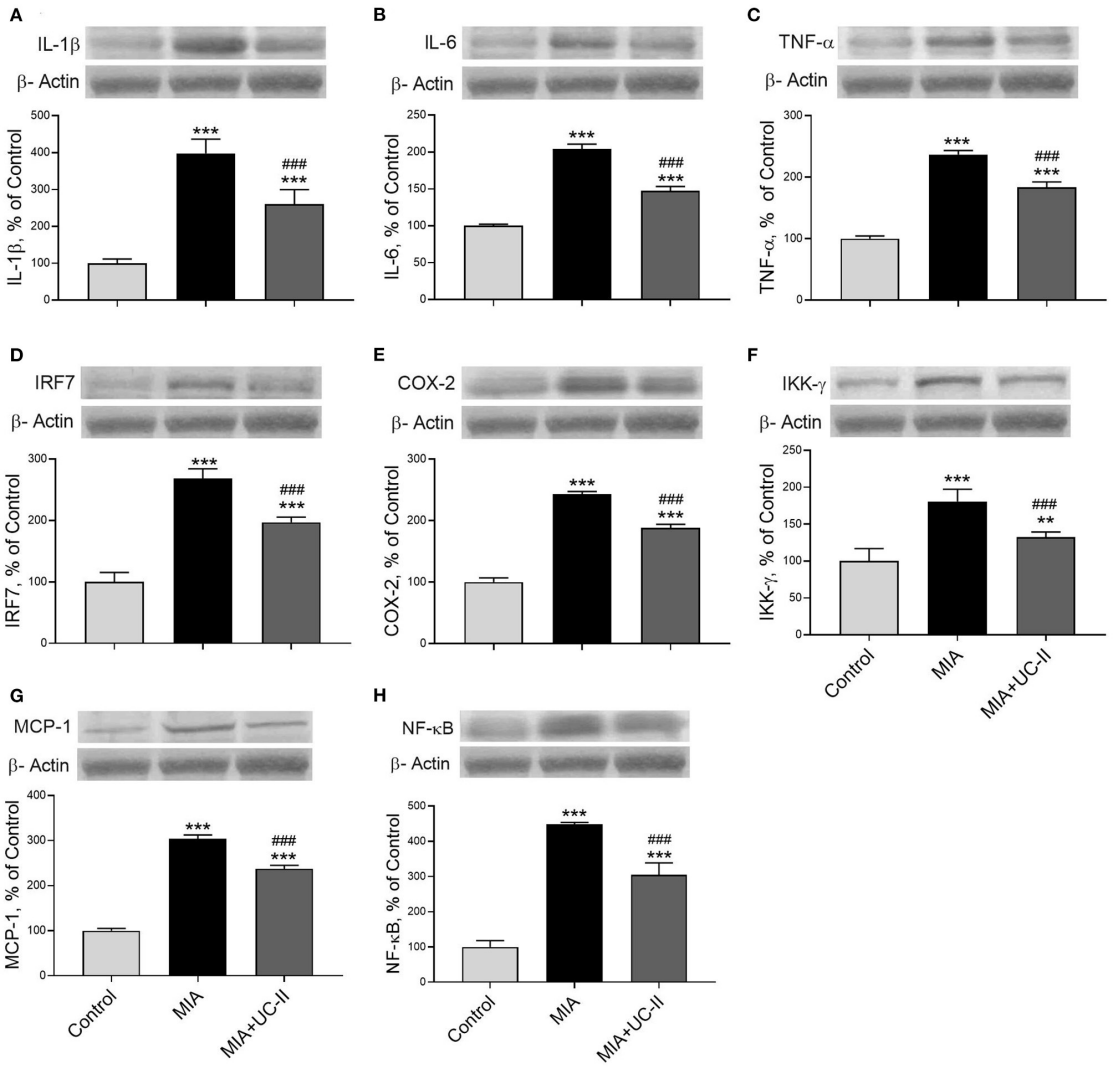
Effect of undenatured type II collagen (UC-II) on knee joint protein expression of interleukin-1β (IL-1β; **A**), interleukin-6 (IL-6; **B**), tumor necrosis factor α (TNF-α; **C**), interferon regulatory factor 7 (IRF7; **D**), cyclooxygenase-2 (COX-2; **E**), nuclear factor-kappa B kinase subunit gamma (IKK-γ; **F**), monocyte chemoattractant protein-1 (MCP-1; **G**), and nuclear factor kappa B (NF-κB; **H**) levels in monosodium iodoacetate (MIA) induced osteoarthritis rats (*n* = 7). The densitometric analysis of the relative intensity according to the control group of the western blot bands was performed with β-actin normalization to ensure equal protein loading. Blots were repeated at least three times (*n* = 3) and representative blots are shown. The bars represent the mean and standard deviation of data. ANOVA and Tukey's *post-hoc test* were used to compare the results among different treatment groups. Statistical significance between groups is shown by: ^**^P < 0.01, ^***^P < 0.001 as compared to control group and, ^###^ *P* < 0.001 as compared to MIA group.

**Figure 7:**
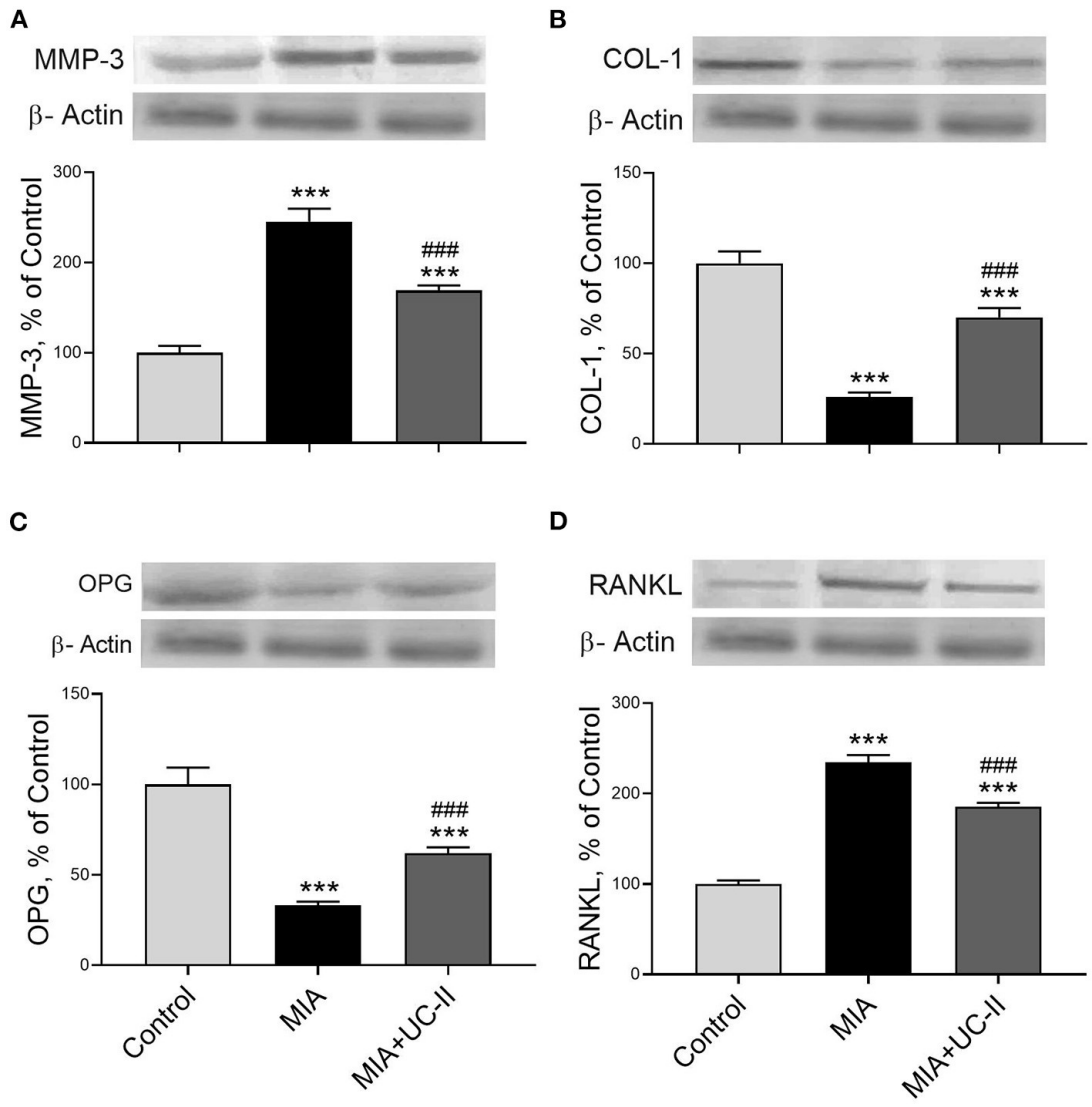
Effect of undenatured type II collagen (UC-II) on knee joint protein expression of metalloproteinase 3 (MMP-3; **A**), collagen type 1 (COL-1; **B**), osteoprotegerin (OPG; **C**), and receptor activator of NF-κB ligand (RANKL; **D**) levels in monosodium iodoacetate (MIA) induced osteoarthritis rats (*n* = 7). The densitometric analysis of the relative intensity according to the control group of the western blot bands was performed with β-actin normalization to ensure equal protein loading. Blots were repeated at least three times (*n* = 3) and representative blots are shown. The bars represent the mean and standard deviation of data. ANOVA and Tukey's *post-hoc test* were used to compare the results among different treatment groups. Statistical significance between groups is shown by: ^***^P < 0.001 as compared to the control group and, ^###^ *P* < 0.001 as compared to MIA group.

OA induction with intraarticular injection of MIA significantly increased the protein levels of IL-1β, IL-6, TNF-α, IRF7, COX-2, IKK-γ, MCP-1, NF-κB, MMP-3, and RANKL in the knee joint tissue (*P* < 0.001 for all; Figures 6, 7) while decreased COL-1 and OPG levels (*P* < 0.001 for all; Figure 7). On the other hand, we observed UC-II supplementation efficiently reduced IL-1β, IL-6, TNF-α, IRF7, COX-2, IKK-γ, MCP-1, NF-κB, MMP-3, and RANKL when compared with untreated MIA rats (*P* < 0.001). We also demonstrated that COL-1 and OPG protein levels increased in knee joint samples of UC-II supplemented rats compared with control rats (*P* < 0.001; Figure 7).

## Discussion

In the current study, we demonstrated that without any toxicological effect on liver and kidney function, UC-II supplementation improves gait measurements, knee joint cartilage deformation, serum inflammatory parameters, and specific OA-related mechanical signal pathways related to MIA-induced OA rats. Consistent with this study, MIA-induced knee joint OA reduced gait measurements due to cartilage damage, as previously reported (21). In addition, joint diameter increased consistently with previous MIA-induced OA studies (22). In the present study, UC-II supplementation improved gait stability, as previously reported (23). The reduction of the claudication severity observed with UC-II supplementation in dogs proves these results (24).

The chondrocyte cell death leads to cartilage deterioration, joint space narrowing, subchondral bone sclerosis, the formation of bone osteophytes (bony outgrowths), and eventually deformity of bone ends (25). Due to these deformities, the Kellgren-Lawrence score used to classify knee OA severity increased with intra-articular MIA injection in this study. As expected, the MIA + UC-II group showed a significant reduction in the Kellgren-Lawrence score due to cartilage deterioration reversing the effects of UC-II (23). There was seen an apparent increase in the Mankin score after the MIA induction in MIA and MIA+UC-II groups. The UC-II supplementation in OA rats slightly improved cartilage microstructure, degeneration, and surface organization that was found to be similar to previous studies (23, 26). Nonetheless, there were no significant differences in Mankin scoring when compared with the untreated rats.

Cartilage and synovium inflammation initiates the formation of OA, and many inflammation factors increase even more with the formation of OA (27). In this study, serum TNF-a, IL-6, CRP, PGE2, and COMP levels increased due to MIA injection, which decreased with UC-II supplementation. Tong et al. (28) demonstrated that using an *in vivo* model of collagen-induced arthritis, ingesting microgram quantities of undenatured type II collagen significantly reduces circulating levels of inflammatory cytokines. The serum TNF-a, CRP, and PGE2 levels probably decreased after UC-II supplementation in this study in relation to decreased pro-inflammatory cytokines (29). Serum COMP levels serve as an early biomarker of arthritis and OA (30). The raised serum COMP levels in the MIA group concur with the others (31). In the current study, the UC-II supplementation decreased serum COMP levels, presumably due to a reduction in cartilage deterioration.

Several studies reported that reactive oxygen species (ROS) production and oxidative stress parameters increased in OA (32). Serum MDA levels also increased with cartilage damage caused by ROS in knee joint OA (33). On the contrary, antioxidant enzymes SOD, CAT, and GSH-Px were reduced in many experimental OA models (34). Similar to previous studies, in this study, OA induction increased serum MDA levels and decreased serum antioxidant enzyme activities. However, UC-II supplementation increased the level of SOD but no other antioxidant enzyme activities. As far as the authors know, there are no previous studies to compare the antioxidant effect of UC-II in the OA model. The increased pro-inflammatory factors, especially TNF-α, IL-1β, phosphorylate the inhibitory κβ proteins (Iκβ) by IκB kinase complex (IKKα, IKKβ, and IKK-γ/NF-κB) to activate the NF-κB located in the cytoplasm (35). The IKK-γ plays a vital role in activating the IKK complex and NF-κB pathway that regulates further cascades in OA progression (36). The released NF-κB provokes the pro-inflammatory cytokines such as TNF-α, IL-1β, and IL-6 while inducing the production of the MCP-1, COX-2, and MMPs (35). The knee joint NF-κB, TNF-α, IL-1β, IL-6, and COX-2 levels in the MIA group increased in our study, consistent with the previous studies (37). However, we showed that UC-II inhibits NF-κB, TNF-α, IL-1β, IL-6, COX-2, and IKK-γ in MIA induced OA rats. Similarly, UC-II supplementation reduced the serum IL-1β, IL-6, and IL-8 and TNF-α levels in collagen-induced arthritis (26, 38). In addition, in the case of osteoarthritis defined by a subclinical immune disorder and the vicious cycle of inflammatory events, UC-II promoted a reduction in inflammation (10). It was reported that UC-II supplementation does not affect the NF-κB levels in human synovial MH7A cells. However, in another study, it was shown that UC-II supplementation regulates the NF-κB pathway-related markers (26, 38).

IRF7 has an anti-inflammatory function in arthritis via the initiating of immunity. Sweeney et al. (39) reported that IRF-7 deficient mice provoked the arthritis symptoms in the passive transfer arthritis model, and IFNβ treatment reduced the arthritis symptoms. In the pathogenesis of OA, the degraded collagen that was occurring from damaged cartilage recognizes as antigen and served by chondrocytes acting as antigen-presenting cells to T cells (5). Therefore, we supposed that the increased IRF7 in MIA groups most likely related to autoimmune reactions in the knee joint cartilage. There are no studies to compare the effects of UC-II on IRF7 after the OA induction.

MCP-1, known as a critical factor of OA initiation, is one of the most common inflammation markers (40). The elevated expression of MCP-1 in the joint tissue results in excess chondrocytes apoptosis that leads to cartilage damage (40). Most probably, UC-II supplementation reduced the MCP-1 levels by suppressing the immune reactions on denatured type II collagen in the knee joint compared to the untreated OA rats. Some of the MMP family enzymes effectively degrade the extracellular matrix, especially type II collagen found in joint cartilage (41). High MMP-3 levels in articular cartilage are considered an effective predictor of OA (42). And the MMP-3 levels in the knee joint increased consistent with the previous MIA-induced OA rat model (41). Although there is a human clinical study reporting that MMP-3 levels in the arthritic knee joint synovium were not affected by UC-II (40 mg/day; 0,57 mg/kg) supplementation (16), we found that UC-II supplementation reduced the MMP-3 levels in knee joint OA rats.

In the late stages of OA, the expression of COL-1 in the subchondral bone matrix raises, but insufficient calcification decreases the bone volume (43). As seen in the present study, COL-1 degradation in the subchondral bone increases due to inflammation in the early stages of rheumatoid arthritis (44) in the MIA group. There is no study found to compare the effect of the UC-II supplementation on knee joint COL-1 levels.

During arthritis, TNF-α, and other inflammation factors continuously stimulate the osteoblasts to produce RANKL, which leads to osteoclast genesis by interacting with RANK located on the osteoclast (45). Unfortunately, decreased OPG levels in the subchondral bone during joint inflammation cannot meet increased RANKL (46). Consequently, the reduced ratio of OPG to RANKL is stressed as a marker of joint damage (47). Similar to Yang et al. (48), after intraarticular MIA injection, the RANKL levels increased in the knee joint of the rats while OPG levels decreased. Although there are no studies to compare the effects of UC-II on RANKL and OPG, after OA induction, the anti-inflammatory effect of the UC-II probably decreased the RANKL level in the knee joint, while the OPG level was increased.

## Conclusion

In conclusion, we reported that the UC-II supplementation effectively reduces the OA symptoms, microstructural deformations of the knee joint, and some catabolic signaling pathways in MIA+UC-II rats. UC-II diminished inflammatory factors by boosting oral tolerance and reduced the inflammatory process. Therefore, we demonstrated that UC-II inhibits inflammatory factors related to the NF-κB signaling pathway and inflammatory mediators such as COX-2 and PGE2. The UC-II supplementation reduced the NF-κB dependent IRF-7 and MCP-1 levels in OA due to its immunomodulatory role. In addition, NF-κB dependent MMP3 pathway and RANKL activation were effectively reduced after the UC-II supplementation.

Consequently, we reported the first time that UC-II prevents NF-κB activation on MIA-induced OA rats. In line with these findings, it was concluded that UC-II supplementation to animals suffering from OA might alleviate joint inflammation by reducing the expression of inflammatory mediators. However, randomized clinical trials are required for further assessments.

## Data Availability Statement

Correspondence and requests for materials should be addressed to K. Sahin.

## Ethics Statement

The Firat University Animal Ethical Committee reviewed and approved (182-2019/126) all procedures.

## Author Contributions

KS, conceptualization, writing – review and editing, KS, NS, CO, MT, and ES, methodology, NS, CO, MT, and ES, formal analysis, investigation, and data curation, KS and ES, writing – original draft preparation, VJ, editing, All the authors read and approved the final manuscript.

## Conflict of Interest

VJ an employee of Lonza Consumer Health (NJ, USA). The remaining authors declare that the research was conducted in the absence of any commercial or financial relationships that could be construed as a potential conflict of interest.
